# On the Problem of Double-Filtering in PPP-RTK

**DOI:** 10.3390/s23010229

**Published:** 2022-12-26

**Authors:** A. Khodabandeh, P. J. G. Teunissen, D. Psychas

**Affiliations:** 1 Department of Infrastructure Engineering, The University of Melbourne, Melbourne 3010, Australia; 2 Department of Geoscience and Remote Sensing, Delft University of Technology, 2628 CN Delft, The Netherlands; 3 GNSS Research Centre, Curtin University, Perth 6845, Australia; 4 European Space Agency (ESA/ESTEC), 2200 AG Noordwijk, The Netherlands

**Keywords:** global navigation satellite system (GNSS), integer ambiguity resolution enabled precise point positioning (PPP-RTK), Kalman filter, double-filtering, time-correlated corrections

## Abstract

To obtain single-receiver Global Navigation Satellite System (GNSS) parameter solutions, the PPP-RTK user-filter combines measurements with time-correlated corrections that are separately computed by the filter of an external provider. The consequence of exercising such double-filtering is that the Kalman filter’s standard assumption of having uncorrelated measurements in time becomes violated. This leads the user-filter to lose its ‘minimum variance’ property, thereby delivering imprecise parameter solutions. The solutions’ precision-loss becomes more pronounced when one experiences an increase in the correction latency, i.e., the delay in time after the corrections are estimated and the time they are applied to the user measurements. In this contribution, we propose a new multi-epoch formulation for the PPP-RTK user-filter upon which both the uncertainty and the temporal correlation of the corrections are incorporated. By a proper augmentation of the user-filter state-vector, the corrections are jointly measurement-updated with the user parameter solutions. Supported by numerical results, the proposed formulation is shown to outperform its commonly used counterpart in the minimum-variance sense.

## 1. Introduction

Integer carrier-phase ambiguity resolution-enabled precise point positioning, PPP-RTK, has the potential to enormously benefit from the state-space packaging of the positioning corrections to reduce their transmission rate, i.e., the frequency with which the corrections are to be provided to single-receiver global navigation satellite system (GNSS) users, see, e.g., [1,2,3,4,5]. However, a reduction in the transmission rate comes at the cost of delivering time-delayed corrections. Consequently, the user is required to time-predict the corrections so as to bridge the gap between the corrections’ generation time and the user positioning time.

Therefore, next to the intrinsic uncertainty brought by the randomness of GNSS measurements, PPP-RTK corrections also inherit extra uncertainty that is associated with their time-prediction [6]. Should the characteristics of such correctional uncertainty, e.g., in terms of corrections’ (co)variance matrices, be made available, the PPP-RTK user would then be in a position to employ rigorous estimation methods so as to achieve the *most-precise* (minimum-variance) parameter solutions [7]. In practice, however, a proper quality description of the corrections is often not provided to the user [8]. This is because the state-space representation of the corrections is aimed at minimizing the amount of information required to be made available. In the absence of such information, the approach commonly taken is to assume that the corrections are precise enough so that they can be treated as *non-random* [9]. The consequence of this practice is that the user estimation method becomes *suboptimal* in the sense that it loses its minimum-variance property and fails to provide the correct quality description of parameter solutions [10].

In our earlier contributions [7,8,9], we showed how the PPP-RTK user can limit the sub-optimality level of their parameter solutions when dealing with a ‘single epoch’ of data. Such single-epoch solutions do benefit from either closed-form expressions of the corrections’ variance matrix or their approximate versions. The topic of the present contribution concerns the scenario where the user aims to estimate their parameters using *multiple epochs* of data in a near real-time manner. The well-known candidate for the real-time computation of such multi-epoch solutions is the Kalman filter [11,12]. In this contribution, we show why existing formulations of the user’s Kalman filter are *misspecified*, and propose an alternative formulation that can largely avoid the precision loss of the user filtered solutions.

The structure of the paper is organized as follows. The problem of double-filtering in PPP-RTK is briefly reviewed in Section 2. The dependence of the user-filter on an external provider-filter is characterized, identifying the factors that lead to a misspecified stochastic model of the user-filter. In Section 3, we discuss potential choices with which the user can weight their time-correlated, ‘corrected’ observation vectors. Simulated examples are given to provide numerical insight into the consequences of such choices. Section 4 is devoted to the new formulation of the user-filter. The recursive structure of the filter is presented and the approximation on which the filter’s optimality is based is highlighted. To numerically demonstrate the superiority of the filter over existing formulations, a single-station PPP-RTK setup [7,13] is employed in Section 5. It is thereby shown how the filter responds to rather high correction *latency*, i.e., the delay in time after the corrections are generated and the time they are applied to the user data. Finally, summary and concluding remarks are provided in Section 6.

The following notation will be used in this paper. The underscore symbol indicates the ‘randomness’ of quantities. Thus, $\underline{x}$ is random, while *x* is not. The hat $\hat{\cdot }$ and check $\overset{ˇ}{\cdot }$ symbols indicate the solutions of unknown parameters. Thus, $\hat{\underline{x}}$ (or $\overset{ˇ}{\underline{x}}$) is a solution of *x*. The subscript t|τ of ${\hat{\underline{x}}}_{t|\tau }$ indicates that ${\hat{\underline{x}}}_{t|\tau }$ is a solution of $x_{t}$ which is obtained based on all the observations collected up to and including the time-instant (epoch) τ. The covariance operator is denoted by Cov(·,·), while the capital *Q* is reserved for (co)variance matrices.

## 2. Optimal Provider-Filter vs. Misspecified User-Filter

We commence with the (linearized) observation equations of a single-receiver PPP-RTK user at epoch *i*

(1) $${\underline{u}}_{i}=B_{i}\;b_{i}+C_{i}\;c_{i}+{\underline{n}}_{i},$$

where the user observation vector ${\underline{u}}_{i}$, together with the zero-mean random noise ${\underline{n}}_{i}$, is linked to the user’s unknown parameter vector $b_{i}$ and the unknown correction vector $c_{i}$ through the full-rank design matrices $B_{i}$ and $C_{i}$. The observation vector ${\underline{u}}_{i}$ may contain the GNSS carrier-phase and pseudorange (code) measurements, with $b_{i}$ containing the position coordinates, carrier-phase ambiguities, receiver clock parameters, and instrumental biases. On the other hand, the correction vector $c_{i}$ may contain estimable forms of satellite orbit and clock parameters, atmospheric parameters, and phase/code biases [14,15,16].

With the sole use of their measurements, the single-receiver user cannot *unbiasedly* determine both the unknown vectors $b_{i}$ and $c_{i}$. In other words, the augmented design matrix $\left[B_{i},C_{i}\right]$ is rank-defect, meaning that part of $b_{i}$ (or of $c_{i}$) has to be held fixed as S-basis so that (1) becomes solvable for *biased* versions of $b_{i}$ and $c_{i}$ [15]. This of course does not suit user positioning. To obtain $b_{i}$ unbiasedly, the PPP-RTK user needs to receive an unbiased solution of the correction vector $c_{i}$ from an external provider, e.g., a network of permanent GNSS stations [1].

### 2.1. Non-Random Corrections as a Basic Principle

For the sake of argument, let us first assume that the correction vector $c_{i}$ is determined by the provider with *no* uncertainty and is made available to the user. Accordingly, the user would correct their observation Equation (1) as follows

(2) $${\underline{u}}_{i}-C_{i}\;c_{i}=B_{i}\;b_{i}+{\underline{n}}_{i}$$

Since matrix $B_{i}$ is of full-column rank, ‘single-epoch’ solutions of the user parameters $b_{i}$ can then be unbiasedly computed. Instead of sticking to one single-epoch of data, the user may improve the precision of their parameter solutions by incorporating the temporal behaviour of some involved parameters. For instance, the user phase ambiguities remain constant in time unless a cycle-slip occurs. The rather stable instrumental biases can be linked in time by a random-walk process [17], while a polynomial dynamic model may be employed to capture the motion of the user position [7]. Such constraints between the user parameter vectors $b_{h}$ (h=i,i+1…) can be expressed by the following dynamic models [18]

(3) $${\underline{o}}_{h}^{b}=b_{h}-\Phi^{b}\;b_{h-1}+{\underline{w}}_{h}^{b},\;\;h=i+1,i+2\ldots$$

where the randomness of the zero-sampled pseudo-observation ${\underline{o}}_{h}^{b}$ is characterized by the process noises ${\underline{w}}_{h}^{b}$. The transition matrix $\Phi^{b}$ links the user parameters between two successive epochs. Thus, $\Phi_{\left(j-i\right)}^{b}=\prod_{h=1}^{j-i}\Phi^{b}$ (j>i) links the parameters from epoch *i* to epoch *j*.

The constraints (3) serve as extra observations to increase the user model’s redundancy, which is, the number of user observations minus the number of the estimable parameters involved. An increase in the redundancy strengthens the user model, thus improving the user parameter solutions [19]. In the context of PPP-RTK, however, the user parameter solutions are to be computed in a near real-time manner, demanding *recursive* estimation methods. The Kalman-filter [11,12] is known to be an *optimal* estimation method to handle such recursive computation in a *minimum-variance* sense. This optimality property relies on a key assumption, however, namely that the corrected observation vectors ${\underline{u}}_{h}-C_{h}\;c_{h}$ (h=i,i+1,…) must be *uncorrelated* in time, and uncorrelated with the process noises ${\underline{w}}_{h}^{b}$ (h=i+1,i+2,…). Likewise, the process noises ${\underline{w}}_{h}^{b}$ must be uncorrelated in time. Provided that the user collects their measurements independently over time, the observations ${\underline{u}}_{h}$ (and therefore their corrected versions ${\underline{u}}_{h}-C_{h}\;c_{h}$) fulfill such an assumption. This serves as a basic principle of PPP-RTK that is commonly exercised in practice [8]. As the will be shown below, however, such a key assumption is violated if the non-random correction vector $c_{h}$ in ${\underline{u}}_{h}-C_{h}\;c_{h}$ is replaced by a ‘random’ correction solution that has been computed by the external provider filter. As a consequence, the underlying model of the PPP-RTK user-filter becomes misspecified, losing its minimum-variance optimality property [10].

### 2.2. Optimal Provider-Filter

The role of a PPP-RTK provider is to estimate the unknown correction vectors $c_{h}$ (h=i,i+1,…) and make the corresponding solutions available to the user. As with the user, the provider formulates their own Kalman filter with the observation equations (at epoch *t*)

(4) $${\underline{y}}_{t}=A_{t}\;c_{t}+{\underline{e}}_{t},\;t=1,2,\ldots ,$$

and the dynamic models

(5) $${\underline{o}}_{t}^{c}=c_{t}-\Phi^{c}\;c_{t-1}+{\underline{w}}_{t}^{c},\;\;t=2,3\ldots$$

where the provider observation vector ${\underline{y}}_{t}$, together with the zero-mean measurement noise ${\underline{e}}_{t}$, are linked to the unknown corrections vector $c_{t}$ through the full-rank design matrix $A_{t}$. The transition matrix $\Phi^{c}$ links the correction vectors between two successive epochs with the zero-sampled pseudo-observation ${\underline{o}}_{t}^{c}$ and time-uncorrelated process noises ${\underline{w}}_{t}^{c}$ (t=2,3…). If the observation vectors ${\underline{y}}_{t}$ (t=1,2,…) are also time-uncorrelated, and uncorrelated with the process noises ${\underline{w}}_{t}^{c}$, the provider would then be in a position to run their optimal minimum-variance filter.

**Figure 1:**
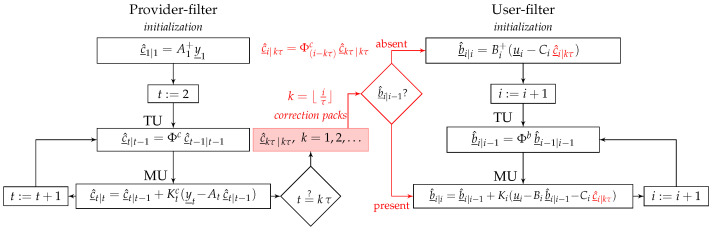
Double-filtering in PPP-RTK. (**Left**): the provider uses ‘time-uncorrelated’ observation vectors ${\underline{y}}_{t}\;\left(t=0,1,\ldots \right)$ to generate and disseminate ‘time-correlated’ correction packs ${\underline{\hat{c}}}_{k\tau \;|\;k\tau }\;\left(k=1,2,\ldots \right)$ every τ seconds. (**Right**): the user picks the ‘most recent’ correction pack to feed their model at epoch *i*. Thus, k=⌊i/τ⌋, where the operator ⌊x⌋ returns the greatest integer less than or equal to *x*. If no previous user solution ${\underline{\hat{b}}}_{i|i-1}$ is present, the user initializes their filter with the corrected observation vector ${\underline{u}}_{i}-C_{i}\;{\underline{\hat{c}}}_{i|k\tau }$. Otherwise, the correction pack is fed into the user MU step.

The three-step structure of the provider-filter is presented in the left-panel of Figure 1. The structure follows from an application of the least-squares principle to (4) and (5), see, e.g., [18]. At the initialization step (first epoch t=1), the provider initializes their filter by the least-squares solution ${\underline{\hat{c}}}_{1|1}=A_{1}^{+}{\underline{y}}_{1}$ with $A_{1}^{+}={\left(A_{1}^{T}Q_{y_{1}}^{-1}A_{1}\right)}^{-1}A_{1}^{T}Q_{y_{1}}^{-1}$, where $Q_{y_{t}}$ denotes the variance matrix of the observation vector ${\underline{y}}_{t}$ (t=1,2,…). The application of the (co)variance propagation law also gives the solution’s error-variance matrix as $Q_{{\hat{c}}_{1|1}}={\left(A_{1}^{T}Q_{y_{1}}^{-1}A_{1}\right)}^{-1}$. At the time-update (TU) step, the correction vector of the upcoming epoch can be time-predicted as ${\underline{\hat{c}}}_{t|t-1}=\Phi^{c}\;{\underline{\hat{c}}}_{t-1|t-1}$ (t=2,3,…), with the error-variance matrix $Q_{{\hat{c}}_{t|t-1}}=\Phi^{c}Q_{{\hat{c}}_{t|t-1}}\Phi^{cT}+Q_{w_{t}^{c}}$, where $Q_{w_{t}^{c}}$ is the variance matrix of ${\underline{w}}_{t}^{c}$. Finally, at the measurement-update (MU) step, the filter makes use of the upcoming observation vector $y_{t}$ to recursively update the correction solution as ${\underline{\hat{c}}}_{t|t}={\underline{\hat{c}}}_{t|t-1}+K_{t}^{c}\left({\underline{y}}_{t}\;-\;A_{t}\;{\underline{\hat{c}}}_{t|t-1}\right)$ with the error-variance matrix $Q_{{\hat{c}}_{t|t}}=\left(I-K_{t}^{c}\;A_{t}\right)Q_{{\hat{c}}_{t|t-1}}$, in which the Kalman gain matrix is given by $K_{t}^{c}=Q_{{\hat{c}}_{t|t-1}}A_{t}^{T}{\left(Q_{y_{t}}+A_{t}Q_{{\hat{c}}_{t|t-1}}A_{t}^{T}\right)}^{-1}$.

### 2.3. Misspecified User-Filter

In contrast to the provider-filter, the user-filter cannot stand on its own and requires the output of the provider-filter, that is, the correction solutions ${\underline{\hat{c}}}_{h|h}$ (h=i,i+1,…). Due to the intrinsic randomness of the provider observations, ${\underline{\hat{c}}}_{h|h}$ is accompanied with an amount of *uncertainty* characterized by the error-variance matrix $Q_{{\hat{c}}_{h|h}}$. With reference to (2), this implies that the random filtered solutions ${\underline{\hat{c}}}_{h|h}$ replace their non-random versions $c_{h}$ to form the user *corrected* observation vectors ${\underline{u}}_{h}-C_{h}\;{\underline{\hat{c}}}_{h|h}$. As the observation vectors ${\underline{u}}_{h}$ are time-uncorrelated, it is the correction solutions ${\underline{\hat{c}}}_{h|h}$ that dictate whether the Kalman filter’s key assumption holds. This is followed by applying the covariance propagation law to corrected observation vectors of any two distinct epochs *j* and *i*, that is

(6) $$\mathrm{Cov}\left({\underline{u}}_{j}-C_{j}{\underline{\hat{c}}}_{j|j}\;,\;{\underline{u}}_{i}-C_{i}{\underline{\hat{c}}}_{i|i}\right)=C_{j}\;Q_{{\hat{c}}_{j|j},\;{\hat{c}}_{i|i}}\;C_{i}^{T},\;j\neq i$$

in which the covariance matrix $Q_{{\hat{c}}_{j|j},\;{\hat{c}}_{i|i}}$ between the correction solutions ${\underline{\hat{c}}}_{j|j}$ and ${\underline{\hat{c}}}_{i|i}$ is shown to read (Appendix A).

(7) $$Q_{{\hat{c}}_{j|j},\;{\hat{c}}_{i|i}}=\left[\prod_{h=i+1}^{j}\left(I-K_{h}^{c}A_{h}\right)\Phi^{c}\right]\;Q_{{\hat{c}}_{i|i}},\;j>i$$

The nonzero covariance matrix above indicates that the user-corrected observation vectors ${\underline{u}}_{h}-C_{h}\;{\underline{\hat{c}}}_{h|h}$ (h=i,i+1,…) are indeed time-correlated, making the user-filter misspecified.

Although the PPP-RTK user-filter is *not* minimum-variance, and thus suboptimal, previous studies have demonstrated that the filter can still deliver successful ambiguity-resolved positioning solutions when the duration of the provider-filter initialization, i.e., the time-difference between the epoch *i* and the initial epoch t=1, becomes sufficiently large (e.g., ∼1 h), as can be seen in, e.g., [6,7,9]. In other words, the filtered solution ${\underline{\hat{c}}}_{i|i}$ can become sufficiently precise so as to neglect its uncertainty relative to that of the user observations, i.e., $Q_{{\hat{c}}_{i|i}}\approx 0$. By making such an approximation, the covariance matrices in (6) and (7) become zero, meaning that the user-filter is expected to deliver minimum-variance solutions. While the duration of the provider-filter initialization can be ensured to be sufficiently long to make that approximation, the corrections *cannot* be *instantaneously* transferred to the user due to the limited data-transmission bandwidth [1]. This is all the more so as PPP-RTK is meant to take advantage of the efficient ‘state-space’ packaging of the corrections to reduce their transmission rate, i.e., the frequency with which the corrections are to be provided to the user. Consequently, PPP-RTK corrections ranging from orbits and clocks to phase biases are stored in, e.g., an Internet server, each having its own sampling period τ. In contrast to RTK for which the correction latency is less than 4 s [20] (i.e., τ≤4), PPP and PPP-RTK state-space corrections are to be provided with higher time-delays. For instance, current PPP real-time service of IGS (https://igs.org/rts/) (accessed on 18 December 2022) disseminates state-space corrections with a typical latency of 5–10 s, see, e.g., [21,22]. In the following, it is shown how such state-space packaging brings additional correctional uncertainty.

The red-box in Figure 1 indicates the ‘correction packs’ ${\underline{\hat{c}}}_{k\tau |k\tau }\;\left(k=1,2,\ldots \right)$ that are generated and stored by the provider every τ s. As a result, the user gains access to the correction packs with a time-delay or ‘latency’, i.e., kτ≤i. The correction latency i−kτ ranges from 0 to τ−1 s. As shown in the right-panel of Figure 1, the user picks the ‘most recent’ correction pack and *time-predicts* the correction as ${\underline{\hat{c}}}_{i|k\tau }=\Phi_{\left(i-k\tau \right)}^{c}\;{\underline{\hat{c}}}_{k\tau |k\tau }$ to feed their model at epoch *i*. Thus, even if the approximation $Q_{{\hat{c}}_{k\tau |k\tau }}\approx 0$ would be plausible, the uncertainty associated with the time-predicted correction ${\underline{\hat{c}}}_{i|k\tau }$ may not be negligible. This is indeed the case when the time-difference i−kτ is considered to be large. To see this, consider the error-variance matrix of ${\underline{\hat{c}}}_{i|k\tau }$ as (Appendix A)

(6) $$Q_{{\hat{c}}_{i|k\tau }}=\Phi_{\left(i-k\tau \right)}^{c}Q_{{\hat{c}}_{k\tau |k\tau }}\Phi_{\left(i-k\tau \right)}^{cT}+\sum_{h=k\tau +1}^{i}\Phi_{\left(i-h\right)}^{c}\;Q_{w_{h}^{c}}\;\Phi_{\left(i-h\right)}^{cT}$$

While the first term in (8) may be considered negligible for a long duration of the provider-filter initialization, the second term increases as the latency or the time-difference i−kτ increases, leading to a misspecified user-filter. Note, for the sake of presentation, that we did not distinguish between each individual correction type (e.g., satellite orbits versus clocks) in Figure 1. We instead only show one common sampling period τ for all correction types. In practice however, each individual correction can, of course, have its own sampling period τ.

The three-step structure of the misspecified user-filter follows, in a way analogous to that of the provider-filter, be it that the role of the correction parameters $c_{t}$ is replaced by the user parameters $b_{i}$, the design matrix $A_{t}$ by $B_{i}$, and the observation vectors ${\underline{y}}_{t}$ by the user corrected observation vectors ${\underline{u}}_{i}-C_{i}\;{\underline{\hat{c}}}_{i|k\tau }$ (Figure 1, right-panel). In contrast to the provider who weights the observation vectors ${\underline{y}}_{t}$ using the inverse of their variance matrix $Q_{y_{t}}$, the user may not have access to the variance matrix of the correction ${\underline{\hat{c}}}_{i|k\tau }$ to properly weight the corrected observation vectors ${\underline{u}}_{i}-C_{i}\;{\underline{\hat{c}}}_{i|k\tau }$. Instead, let us assume that the user takes a given inverse-weight matrix ${\bar{Q}}_{u_{i}}$ to replace its provider-counterpart $Q_{y_{t}}$. As shown in [10], the misspecified user-filter would then report *incorrect* error-variance matrices as

(9) $$\begin{array}{cccc} \left(\mathrm{initialization}\right): & {\bar{Q}}_{{\hat{b}}_{i|i}} & = & {\left(B_{i}^{T}{\bar{Q}}_{u_{i}}^{-1}B_{i}\right)}^{-1},\;i:=i+1\;\\ \left(\mathrm{time}-\mathrm{update}\right): & {\bar{Q}}_{{\hat{b}}_{i|i-1}} & = & \Phi^{b}{\bar{Q}}_{{\hat{b}}_{i|i-1}}\Phi^{bT}+Q_{w_{i}^{b}},\;\\ \left(\mathrm{measurement}-\mathrm{update}\right): & {\bar{Q}}_{{\hat{b}}_{i|i}} & = & \left(I-K_{i}\;B_{i}\right){\bar{Q}}_{{\hat{b}}_{i|i-1}} \end{array}$$

where the Kalman gain matrix is evaluated as $K_{i}={\bar{Q}}_{{\hat{b}}_{i|i-1}}B_{i}^{T}{\left({\bar{Q}}_{u_{i}}+B_{i}{\bar{Q}}_{{\hat{b}}_{i|i-1}}B_{i}^{T}\right)}^{-1}$. Here and in the following, the $\bar{\cdot }$-symbol over the Capital *Q* is used to distinguish incorrect variance matrices. Thus, not only does the user-filter deliver suboptimal solutions, but it also fails to provide the correct quality description of the user parameter solution ${\underline{\hat{b}}}_{i|i}$. In the following, we discuss a choice of the inverse-weight matrix ${\bar{Q}}_{u_{i}}$ which is often adopted in practice.

## 3. On the Choice of the Inverse-Weight Matrix ${\bar{Q}}_{u_{i}}$

In the previous section, it was shown that the main rinput of the user-filter, i.e., the corrected observation vectors ${\underline{u}}_{h}-C_{h}\;{\underline{\hat{c}}}_{h|k\tau }$ (h=i,i+1,…), are time-correlated cf. (6), prohibiting the *recursive* computation of the *most precise* parameter solutions. By adopting the inverse-weight matrix ${\bar{Q}}_{u_{h}}$ for ${\underline{u}}_{h}-C_{h}\;{\underline{\hat{c}}}_{h|k\tau }$, however, the user can still recursively compute suboptimal parameter solutions using their misspecified filter (cf. Figure 1). Relying on the assumption that the filtered corrections ${\underline{\hat{c}}}_{k\tau |k\tau }$ are sufficiently precise to make the approximation $Q_{{\hat{c}}_{k\tau |k\tau }}\approx 0$, one may choose ${\bar{Q}}_{u_{h}}$ in a way to limit the sub-optimality level of the parameter solutions. For instance, if the uncertainty involved in the time-prediction of the *time-delayed* corrections ${\underline{\hat{c}}}_{h|k\tau }$ can be ignored (i.e., the second term in (8) is neglected), the inverse-weight matrix ${\bar{Q}}_{u_{h}}$ can then be set to the variance matrix of the user observations ${\underline{u}}_{h}$, that is

(10) $$\begin{array}{cc} \mathrm{Case}1: & {\bar{Q}}_{u_{h}}:=Q_{u_{h}},\;h=i,i+1,\ldots \end{array}$$

For this case, the user only takes the variance matrix of their own data, i.e., $Q_{u_{h}}$, for the weighting of the corrected data ${\underline{u}}_{h}-C_{h}\;{\underline{\hat{c}}}_{h|k\tau }$. In other words, the external corrections ${\underline{\hat{c}}}_{h|k\tau }$ are considered sufficiently precise to be treated as *non-random*, the scenario that is commonly exercised in practice [7,8]. This is because the corrections’ error variance matrix $Q_{{\hat{c}}_{k\tau |k\tau }}$ is often not provided to the user. After all, the purpose of using state-space PPP-RTK corrections is to minimize the amount of information to be transmitted to the user [1]. The following example shows the consequence of this choice, i.e., Case 1 (10).

**Example 1.**  *To give primary numerical insight into the consequence of choosing (10), consider a single-receiver user with a known location who is tracking the L1/L2 dual-frequency code data of a pair of satellites to determine the corresponding single-differenced (SD) slant ionospheric delay over 100 epochs with 1 Hz measurement sampling-rate. The user is given a satellite clock offset- and rate-corrections every τ=10 s. Thus, the correction latency ranges from 0 to 9 s. In this simulation example, the filtered corrections ${\underline{\hat{c}}}_{k\tau |k\tau }$ are assumed and simulated to be non-random. Thus, $Q_{{\hat{c}}_{k\tau |k\tau }}=0$. However, the user still has to time-predict the satellite clock corrections ${\underline{\hat{c}}}_{i|k\tau }$ at every epoch i to compute their filtered ionospheric solution ${\underline{\hat{b}}}_{i|i}$. The time-behaviour of the undifferenced ionospheric delays is modelled by a random-walk process with a standard-deviation of 1 $\mathrm{mm}/\sqrt{s}$, whereas the undifferenced satellite clocks follow a constant-velocity dynamic model with a standard-deviation of 1 $cm/\sqrt{s^{3}}$, as can be seen in, e.g., [6]. The standard-deviation of the undifferenced code data is set to 20 cm.*

**Figure 2:**
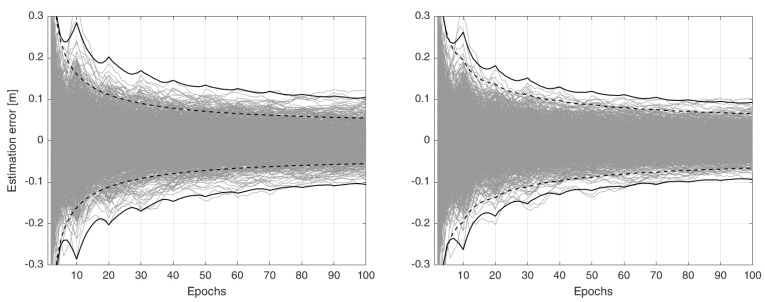
Time–series of the ionospheric estimation–error $b_{h}-{\underline{\hat{b}}}_{h|h}$ (h=1,…,100) of Example 1 (grey lines). Given 1000 normally distributed samples, the misspecified user-filter computes filtered ionospheric solutions under two cases. (**Left**): Case 1 (10) in which the corrections are considered non-random. (**Right**): State-augmentation (12) in which the time-correlation of the corrections is modelled by an exponential auto-correlation function. The incorrect 99.9% confidence intervals (dashed lines), reported by the user-filter, are compared with their correct versions (black solid lines).

*To measure how the misspecified user filter under Case 1 performs, 1000 normally distributed samples of both the correction and user data are simulated over the fixed 100 epochs. The corresponding samples of the ionospheric estimation-error $b_{h}-{\underline{\hat{b}}}_{h|h}$ (h=1,…,100), i.e., the difference between the true simulated ionospheric parameter and its filtered solution, are shown in the left-panel of Figure 2 (grey lines). The black solid lines indicate the associated 99.9% confidence-intervals, whereas the dashed lines represent the corresponding* incorrect *confidence-intervals which are reported by the user filter. As the number of user epochs increases, the dispersion of the estimation-error becomes smaller, showing that the precision of the user filtered solution improves. However, more than 100 epochs of data are required to have the absolute value of the 99.9% confidence-intervals smaller than 1 dm. On the contrary, the filter reports rather optimistic confidence-intervals. The considerable gap between the correct and incorrect confidence-intervals of the user parameter solutions is due to the choice made in (10), i.e., both the variance and time-correlation of the time-predicted corrections are ignored.*

*As the random corrections ${\underline{\hat{c}}}_{i|k\tau }$ make the observation vectors ${\underline{u}}_{i}-C_{i}\;{\underline{\hat{c}}}_{i|k\tau }$ time-correlated, one may employ the technique of* first-order Markov state-augmentation *to handle time-correlated measurements of the Kalman filter, as can be seen in, e.g., [23] (p. 180) or [24]. In that technique, the time-correlation of the corrections is ‘approximated’ by an exponential auto-correlation function [18], that is (compare with (7))*

(11) $${\bar{Q}}_{{\hat{c}}_{j|j},\;{\hat{c}}_{i|i}}:={\bar{Q}}_{c}\;e^{-\frac{1}{\alpha }\left|j-i\right|},\;\forall \;i,j$$

*where the matrix ${\bar{Q}}_{c}$ is to capture the uncertainty of the correction at every epoch. Next to the inverse-weight matrix ${\bar{Q}}_{u_{h}}$ in (10), the state-augmentation technique incorporates the corrections’ variance and time-correlation via the matrices ${\bar{Q}}_{{\hat{c}}_{j|j},\;{\hat{c}}_{i|i}}$ to weight both the* uncorrected *user observations ${\underline{u}}_{h}$ and the time-predicted corrections ${\underline{\hat{c}}}_{h|k\tau }$. The coefficient α governs the magnitude of the correlation between the observation vectors. The larger the coefficient α, the larger the time-correlation is assumed between the observation vectors. To run their filter in recursive form, the user would need to make the following modifications [18]*

(12) $$B_{h}\mapsto \left[B_{h},C_{h}\right],\;b_{h}\mapsto \left[\begin{array}{c} b_{h}\\ a_{h} \end{array}\right],\;\Phi^{b}\mapsto \left[\begin{array}{cc} \Phi^{b} & 0\\ 0 & e^{-\frac{1}{\alpha }}I \end{array}\right],\;{\bar{Q}}_{u_{h}}\mapsto \left[\begin{array}{cc} Q_{u_{h}} & 0\\ 0 & {\bar{Q}}_{c} \end{array}\right],\;Q_{w_{h}^{b}}\mapsto \left[\begin{array}{cc} Q_{w_{h}^{b}} & 0\\ 0 & {\bar{Q}}_{c}\left(1-e^{-\frac{2}{\alpha }}\right) \end{array}\right]$$

*for epochs h>i, where the notation ‘A↦B’ means ‘replace A by B’. Thus, the user state-vector $b_{h}$ is* augmented *with the parameter vector $a_{h}$ whose process noises’ time-correlation exponentially decays over time. For the user initial epoch h=i, the filter is initialized by the augmented state-vector ${\left[{\underline{\hat{b}}}_{i|i}^{T},{\hat{a}}_{i|i}^{T}\right]}^{T}$, with ${\underline{\hat{b}}}_{i|i}=B_{i}^{+}\left({\underline{u}}_{i}-C_{i}{\underline{\hat{c}}}_{i|k\tau }\right)$ and ${\hat{a}}_{i|i}=0$. Thus, the initial user solution ${\underline{\hat{b}}}_{i|i}$ is identical to that of Case 1 (10). For the upcoming epochs h>i, however, the filter aims to capture the randomness of the corrections by updating the solutions ${\hat{\underline{a}}}_{h|h}$ over time.*

*To see the extent to which the state-augmentation technique can alleviate the effect of the time-correlated corrections, we evaluate the ionospheric estimation errors using the coefficients α=50 and ${\bar{Q}}_{c}=0.02$ m${}^{2}$. These coefficients are empirically chosen so as to approximate the time-correlation of the corrections. The results, together with their correct and incorrect 99.9% confidence intervals, are depicted in the right-panel of Figure 2. As shown, the precision of the user filtered solution slightly improves, that is, the absolute value of its 99.9% confidence-intervals reaches 1 dm after 75 epochs. The gap between the correct and incorrect confidence-intervals also becomes smaller.*

As shown in Example 1, treating the time-delayed PPP-RTK corrections as non-random leads to an incorrect and optimistic quality description of the user parameter solutions. Alternatively, the state-augmentation technique may be used to ‘approximate’ both the variance and time-correlation of the corrections via the exponential auto-correlation function (11). Such a technique does, however, *not* take advantage of the information contained in the corrections’ dynamic model (5), thereby discarding the structure of the error-variance matrix (8). We now consider another case that incorporates (8) into the user-filter. As stated before, the approximation $Q_{{\hat{c}}_{k\tau |k\tau }}\approx 0$ may hold by letting the duration of the provider-filter initialization be sufficiently long. The substitution of $Q_{{\hat{c}}_{k\tau |k\tau }}=0$ into (8) gives an inverse-weight matrix ${\bar{Q}}_{{\hat{c}}_{i|k\tau }}$ for weighting the time-predicted corrections ${\underline{\hat{c}}}_{i|k\tau }$. Accordingly, the user inverse-weight matrix ${\bar{Q}}_{u_{h}}$ in (10) is modified as follows

(13) $$\begin{array}{cc} \mathrm{Case}2: & {\bar{Q}}_{u_{h}}:=Q_{u_{h}}+C_{i}\;{\bar{Q}}_{{\hat{c}}_{i|k\tau }}C_{i}^{T},\;h=i,i+1,\ldots ,\;\\ & \;\mathrm{with}\;{\bar{Q}}_{{\hat{c}}_{i|k\tau }}=\sum_{h=k\tau +1}^{i}\Phi_{\left(i-h\right)}^{c}\;Q_{w_{h}^{c}}\;\Phi_{\left(i-h\right)}^{cT} \end{array}$$

Thus, the inverse-weight matrix ${\bar{Q}}_{{\hat{c}}_{i|k\tau }}$ only considers the second term in (8), i.e., the uncertainty due to the time-prediction of the corrections. Although the randomness of PPP-RTK corrections is taken into account under Case 2 (13), their nonzero time-correlation (7) is still dismissed. As with Case 1 (10), such a time-correlation dismissal is required to run the user-filter in its recursive form. In the next section, we show how Case 2 can be generalized so as to account for the corrections’ time-correlation (7), yet allowing the recursive estimation of user parameters.

## 4. User-Filter with Correctional Update

To date, the focus has been restricted to the choice of the user inverse-weight matrix ${\bar{Q}}_{u_{h}}$ that can be made in Cases 1 and 2. Depending on how the user weights their corrected observation vectors ${\underline{u}}_{h}-{\underline{\hat{c}}}_{h|k\tau }$ (h=i,i+1,…) through ${\bar{Q}}_{u_{h}}$, their misspecified filter can be recursively run in accordance with the right-panel of Figure 1. In both Cases 1 and 2, however, the time-predicted corrections ${\underline{\hat{c}}}_{h|k\tau }$ do *not* benefit from the information contained in the user observations ${\underline{u}}_{h}$. In other words, the corrections are merely derived from the correction packs ${\underline{\hat{c}}}_{k\tau |k\tau }$, that is ${\underline{\hat{c}}}_{h|k\tau }=\Phi_{\left(h-k\tau \right)}^{c}\;{\underline{\hat{c}}}_{k\tau |k\tau }$ over the epochs h=i,…,(k+1)τ−1.

As with the first-order Markov state-augmentation (12), the idea is to augment the user state-vector, with a difference, so that the correction parameter vector $c_{h}$ now replaces the parameter vector $a_{h}$. Thus, instead of approximating the corrections’ time-correlation by an exponential auto-correlation function (11), the goal is now to directly incorporate the corrections’ dynamic model (5) into the user-filter. To this end, we again assume that the approximation $Q_{{\hat{c}}_{k\tau |k\tau }}\approx 0$ holds. The initialization of the user-filter is then executed through the following modifications

(14) $$\begin{array}{cc} \mathrm{Case}3\;\left(\mathrm{initialization}\right): & b_{i}\mapsto \left[\begin{array}{c} b_{i}\\ c_{i} \end{array}\right],\;B_{i}\mapsto \left[\begin{array}{cc} B_{i} & C_{i}\\ 0 & I \end{array}\right],\;\left({\underline{u}}_{i}-C_{i}\;{\underline{\hat{c}}}_{i|k\tau }\right)\mapsto \left[\begin{array}{c} {\underline{u}}_{i}\\ {\underline{\hat{c}}}_{i|k\tau } \end{array}\right],\;{\bar{Q}}_{u_{i}}\mapsto \left[\begin{array}{cc} Q_{u_{i}} & 0\\ 0 & {\bar{Q}}_{{\hat{c}}_{i|k\tau }} \end{array}\right] \end{array}$$

where the inverse-weight matrix ${\bar{Q}}_{{\hat{c}}_{i|k\tau }}$ follows from that of (13). In contrast to Cases 1 and 2 in which the corrections are merely time-updated from ${\underline{\hat{c}}}_{k\tau |k\tau }$, we now let the user-filter, next to the time-updating, also *measurement-update* the corrections ${\underline{\hat{c}}}_{i|k\tau }$. We therefore employ the discriminating notation $\overset{ˇ}{\cdot }$ (instead of $\hat{\cdot }$) to denote the user-augmented parameter solutions ${\left[{\underline{\overset{ˇ}{b}}}_{i|i}^{T},\;{\underline{\overset{ˇ}{c}}}_{i|i}^{T}\right]}^{T}$. During the period in which no newer correction pack is available, the user-filter recursively performs the time- and measurement-updates via the following settings

(15) $$\begin{array}{cc} \mathrm{Case}3\;\left(\mathrm{TU}-\mathrm{MU}\right): & \left({\underline{u}}_{h}-C_{h}\;{\underline{\hat{c}}}_{h|k\tau }\right)\mapsto {\underline{u}}_{h},\;{\bar{Q}}_{u_{h}}\mapsto Q_{u_{h}},\;B_{h}\mapsto \left[B_{h},\;C_{h}\right],\;\\ & \Phi^{b}\mapsto \left[\begin{array}{cc} \Phi^{b} & 0\\ 0 & \Phi^{c} \end{array}\right],\;Q_{w_{h}^{b}}\mapsto \left[\begin{array}{cc} Q_{w_{h}^{b}} & 0\\ 0 & Q_{w_{h}^{c}} \end{array}\right],\;\;h=i+1,\ldots ,\left(k+1\right)\tau -1 \end{array}$$

**Figure 3:**
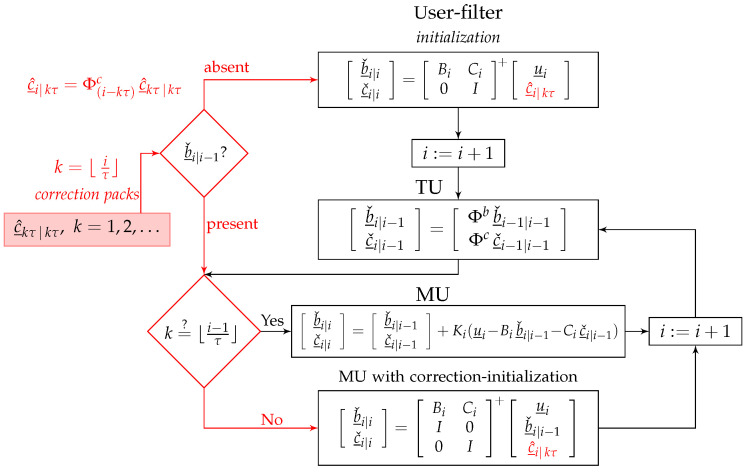
The proposed PPP-RTK user-filter with a correctional update. In the absence of user solution ${\underline{\overset{ˇ}{b}}}_{i|i-1}$, the user picks the ‘most recent’ corrections ${\underline{\hat{c}}}_{i|k\tau }$ and initializes their filter via the augmented observation vector ${\left[{\underline{u}}_{i}^{T},\;{\underline{\hat{c}}}_{i|k\tau }^{T}\right]}^{T}$. The filter does not only deliver the user parameter solution ${\underline{\overset{ˇ}{b}}}_{i|i}$, but also recursively updates the corrections as ${\underline{\overset{ˇ}{c}}}_{i|i}$ (i:=i+1). At every MU step, if a newer correction pack ${\underline{\hat{c}}}_{k\tau |k\tau }$ is made available by the provider (i.e., if $k>\lfloor \frac{i-1}{\tau }\rfloor$), then the user-filtered corrections ${\underline{\overset{ˇ}{c}}}_{i|i}$ are initialized by ${\underline{\hat{c}}}_{i|k\tau }$.

At epoch i=(k+1)τ, when the new correction pack ${\underline{\hat{c}}}_{(k+1)\tau |(k+1)\tau }$ is made available by the provider, the new time-predicted corrections ${\underline{\hat{c}}}_{i|(k+1)\tau }$ are to replace the existing user-filtered corrections ${\underline{\overset{ˇ}{c}}}_{i|i}$. This follows from the approximation $Q_{{\hat{c}}_{(k+1)\tau |(k+1)\tau }}\approx 0$ (k=1,2,…), namely that the new corrections ${\underline{\hat{c}}}_{i|(k+1)\tau }$ contain all the information contained in the user-filtered corrections ${\underline{\overset{ˇ}{c}}}_{i|i}$. The structure of our proposed user-filter is presented in Figure 3. As shown, the user-filtered corrections ${\underline{\overset{ˇ}{c}}}_{i|i}$ have to be initialized and replaced by ${\underline{\hat{c}}}_{i|k\tau }$ every time a newer correction pack ${\underline{\hat{c}}}_{k\tau |k\tau }$ is made available. Note that such a filter is still misspecified, thus delivering suboptimal solutions. However, its sub-optimality level is only dictated by the extent to which the error-variance matrix $Q_{{\hat{c}}_{k\tau |k\tau }}$ is different from zero. This is in marked contrast to Cases 1 and 2, whose solutions’ loss of precision is also driven by the correction latency i−kτ. To compare the performance of the proposed filter with those of Cases 1 and 2, let us again consider the problem in Example 1.

**Figure 4:**
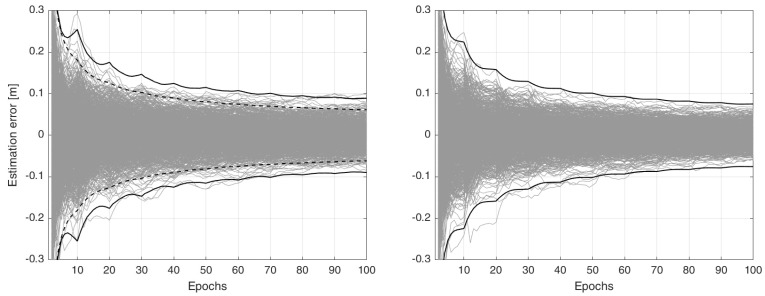
Time–series of the ionospheric estimation-error $b_{h}-{\underline{\hat{b}}}_{h|h}$ (h=1,…,100) of Example 2 (grey lines). Given 1000 normally distributed samples, the misspecified user-filter computes filtered ionospheric solutions under two cases. (**Left**): Case 2 (13), in which only the variance of the corrections is considered. (**Right**): Case 3 (Figure 3), in which both the variance and time-correlation of the corrections are incorporated by the proposed filter. The incorrect 99.9% confidence intervals (dashed lines), reported by the user-filter, are compared with their correct versions (black solid lines).

**Example 2**  (Continuation of Example 1)**.** *Given the same 1000 normally distributed samples of both the correction and user data in Example 1, we evaluate the ionospheric estimation-errors $b_{h}-{\underline{\hat{b}}}_{h|h}$ (h=1,…,100) by the user-filter using the settings om Case 2 (13) and our proposed settings as outlined in Figure 3. The corresponding results are presented in Figure 4. In comparison to the results in Figure 2, Case 2 outperforms Case 1, showing a similar performance to that of the state-augmentation technique (12), i.e., it requires at least 70 epochs to have solutions more precise than 1 dm (with 99.9% confidence). Similar to Case 1 and the state-augmentation technique, there is also a gap between the correct and incorrect confidence intervals. The proposed filter does, however, deliver the most precise results in the sense that the absolute value of their 99.9% confidence-intervals becomes smaller than 1 dm after 50 epochs. At the same time, there is no gap between the correct and reported confidence intervals. This is because the correction packs ${\hat{c}}_{k\tau |k\tau }$ are simulated in a way to be non-random, $Q_{{\hat{c}}_{k\tau |k\tau }}=0$. In the next section, the performance of the proposed user-filter is studied for a real-world GNSS data-set for which the equality $Q_{{\hat{c}}_{k\tau |k\tau }}=0$ does* not *hold. Instead, the filter has to rely on the approximate counterpart $Q_{{\hat{c}}_{k\tau |k\tau }}\approx 0$.*

## 5. A Single-Station PPP-RTK Example

In this section, we make use of a Galileo dual-frequency (E1/E5a) data-set to study the positioning performance of the misspecified user-filter for three variants: Case 1 (10), Case 2 (13), and Case 3 (Figure 3). The data-set was collected with a 1 Hz sampling-rate on 21 January 2022 by two permanent GNSS stations: CUT0 and UWA0, both located in Western Australia. The precise orbital corrections are a priori applied to the data.

**Figure 5:**
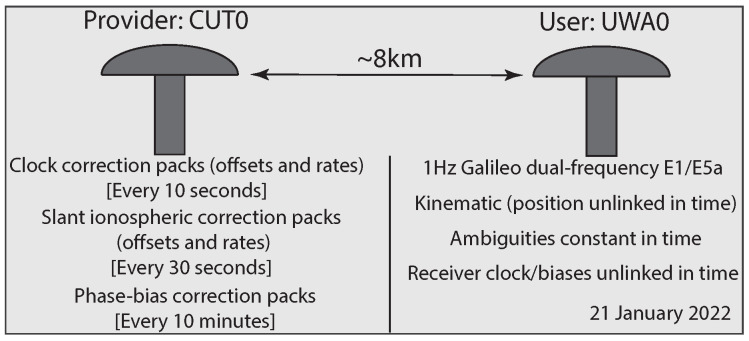
Single-station PPP-RTK setup for the experimental data-set. The temporal behaviour of the satellite clocks and ionospheric delays is modelled by a constant-velocity dynamic model with process noises’ standard deviations of 3 mm/$\sqrt{s^{3}}$ and 1 mm/$\sqrt{s^{3}}$, respectively. The satellite phase biases are assumed to follow a random-walk (constant-state) process with a standard deviation of 0.01 cyc./$\sqrt{s}$.

State-space corrections (i.e., clock, ionospheric and phase-bias corrections) are generated via a single-station PPP-RTK setup [7,13]. The setup is visualized in Figure 5. Station CUT0 serves as correction-provider, whereas the station UWA0 serves as a user that is approximately 8 km away from the provider. To emphasize the performance of our filter formulation (Case 3) in handling time-delayed corrections, we consider rather high correction latencies. Accordingly, the clock correction packs are assumed to be made available to the user every 10 s, ionospheric correction packs every 30 s, and phase-bias correction packs every 10 min. In order to make the approximation $Q_{{\hat{c}}_{k\tau |k\tau }}\approx 0$ plausible, the provider sends the corrections only after 1 h from the time of their filter-initialization. The user then time-predicts each correction and runs their filter over 20 min (1200 epochs). To form their observation variance matrix, the user employs the sinusoidal satellite elevation-weighting strategy with the zenith-referenced standard-deviations of 20 cm and 2 mm for their undifferenced code and phase measurements, respectively.

**Figure 6:**
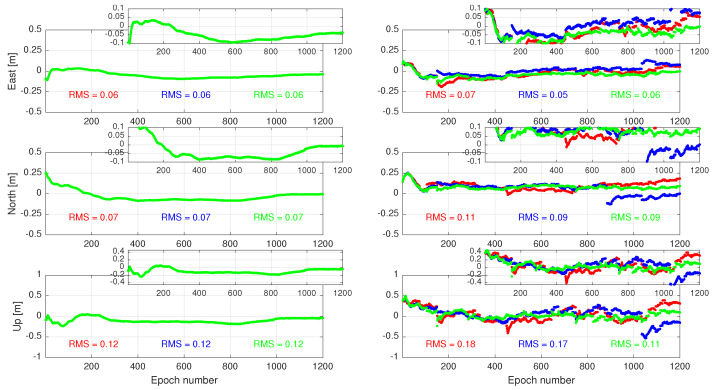
Time-series of the **ambiguity-float** positioning errors in the East–North–Up directions when the user experiences no correction latency (**left**) and when the correction packs are provided with a time-delay (**right**). The results of Cases 1, 2, and 3 are indicated in red, blue, and green, respectively.

Over the fixed 1200 epochs, we consider both the ambiguity-float and -fixed positioning performance of the user. To show the role played by the correction latency, we also compare the user positioning results with those obtained with *zero* latency. Let us first focus on the ambiguity-float results, i.e., before resolving the user float ambiguities to their integers. The results are presented in Figure 6. The left-panel corresponds to the case where no correction latency is experienced by the user. For the zero-latency case, the results of Cases 1 and 2 *coincide* with those of Case 3 (green lines). This can be understood as follows. Recall that Case 1 (red lines) treats the corrections as non-random, and Case 2 (blue lines) models the variance of the corrections, while Case 3 models both the variance and time-correlation of the corrections. As both Cases 2 and 3 rely on the approximation $Q_{{\hat{c}}_{k\tau |k\tau }}\approx 0$, they deliver results identical to those of Case 1 in the case of zero latency (i.e., when i=kτ). Thus, the underlying difference of these three cases only lies in the way of correctional uncertainty due to the fact that the latency is handled.

The effect of nonzero correction latency is shown in the right-panel of Figure 6. Since i≥kτ, the user has to time-predict the corrections, thereby requiring that it take the associated correctional uncertainty into account. As Case 1 does not consider such uncertainty, it delivers positioning results with the largest root-mean-squared (RMS) errors in all the three East–North–Up directions. For the horizontal components, Case 2 delivers similar results to those of Case 3 in an RMS sense. However, Case 3 experiences less discontinuities in its positioning results than Case 2. Such discontinuities are owed to the *periodic* increase in the latency that it varies from 0 to τ−1 seconds for every correction pack.

**Figure 7:**
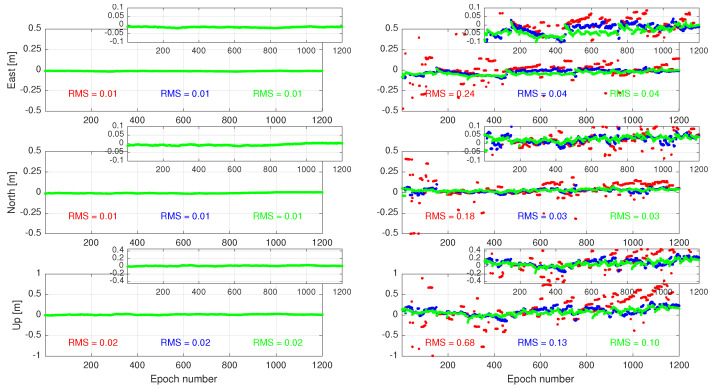
Time-series of the **ambiguity-fixed** positioning errors in East–North–Up directions when the user experiences no correction latency (**left**) and when the correction packs are provided with a time-delay (**right**). The results of Cases 1, 2, and 3 are indicated in red, blue, and green, respectively.

Let us now turn our attention to the user ambiguity-fixed results, i.e., after mapping the float ambiguities to their integers. At every epoch, full ambiguity resolution using the integer least-squares estimation is conducted *without* an extra ambiguity validation procedure [25]. The results are presented in Figure 7. As before, both the zero-latency (left-panel) and nonzero-latency (right-panel) cases are considered. While the three cases 1, 2, and 3 perform the same when the user receives corrections with no time-delay, their performances become distinct when treating time-delayed corrections. Case 1 (red lines) exhibits several large ambiguity-resolved positioning errors (≥1 dm) in all three East–North–Up directions, leading to large positioning RMS errors. In other words, the corresponding ambiguity success-rate is not sufficiently high to deliver successful ambiguity-resolved positioning solutions. On the contrary, Cases 2 and 3 outperform Case 1 in the RMS sense. Similar to their ambiguity-float counterparts, the RMS errors of Cases 2 and 3 are the same for the horizontal components.

**Figure 8:**
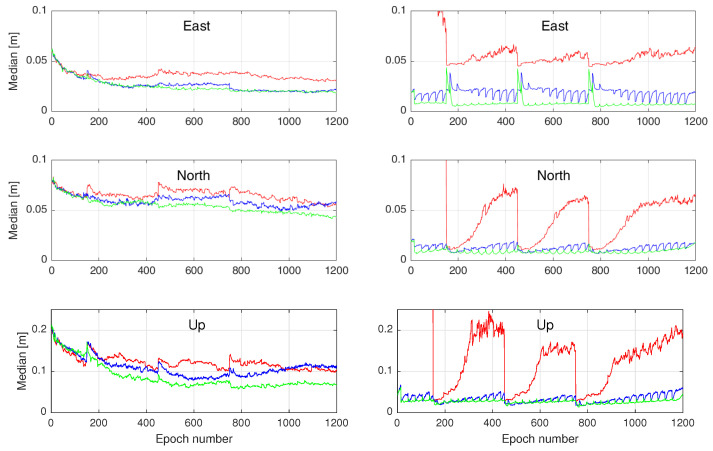
Medians (50% percentiles) of the absolute positioning errors corresponding to 300 user-filter realizations before (**left**) and after (**right**) ambiguity-fixing in the East–North–Up directions when the correction packs are provided with a time-delay (cf. Figure 5). The results of Cases 1, 2, and 3 are indicated in red, blue, and green, respectively.

Observing the results in Figure 6 and Figure 7, one may be inclined to conclude that the performance of the proposed user-filter (Case 3) is the same as that of Case 2. From a computational point of view, Case 2 seems to be more attractive as it does not need the augmentation of the user state-vector with the corrections. This is in contrast to the formulation of Case 3 which needs to recursively update both the user and correction parameter solutions. One should, however, remark that those results only represent one *realization* of the user-filter’s parameter solutions. In order to infer the overall performance of the user-filter under the formulations offered by Cases 1, 2, and 3, one needs to instead consider the *distribution* of the user parameter solutions. To that end, we generate 300 different realizations of the results by shifting the user-filter starting epoch *i* every 15 s. The time-series of the medians (i.e., 50% percentiles) of these realizations are presented in Figure 8 for both the user ambiguity-float (left) and -fixed (right) cases. The medians of the positioning errors corresponding to Cases 2 and 3 are shown to be considerably smaller than those of Case 1. The results also indicate that Case 3 outperforms Case 2 as it, on average, delivers smaller medians of the positioning errors. Note also the presence of periodic jumps of the medians for all the three cases. This behaviour is due to the periodic nature of the correction latencies that vary from zero to τ−1 s for each correction pack. It is, however, observed that Case 3 exhibits smaller periodic jumps. This is because Case 3 accounts for the uncertainty of the time-delayed corrections by modelling their variance and time-correlation through their dynamic model (5).

## 6. Conclusions and Outlook

In this contribution, a new multi-epoch formulation for the PPP-RTK user-filter was proposed (Figure 3). Under the proposed formulation, the user-filter state-vector $b_{h}$ is augmented with the correction parameter vector $c_{h}$ as ${\left[b_{h}^{T},c_{h}^{T}\right]}^{T}$, thereby allowing the corrections to be *jointly* measurement-updated with the user parameter solutions.

It was shown why the user-filter always remains misspecified, and therefore, *suboptimal* in the minimum-variance sense, no matter which formulation is adopted. Since the PPP-RTK provider has the freedom to disseminate their filtered corrections ${\underline{\hat{c}}}_{k\tau |k\tau }$ only after a sufficiently long period from the time of their filter-initialization, the user can, however, benefit from the approximation $Q_{{\hat{c}}_{k\tau |k\tau }}\approx 0$ to limit the sub-optimality level of their filter. Instead of current formulations in which the time-correlated corrected observation vectors ${\underline{u}}_{h}-C_{h}\;{\underline{\hat{c}}}_{h|k\tau }$ (h=i,i+1,…) serve as input of the user-filter, our proposed formulation treats the corrections ${\underline{\hat{c}}}_{h|k\tau }$ as additional observation vectors and works with the augmented observation vector ${\left[{\underline{u}}_{h}^{T},\;{\underline{\hat{c}}}_{h|k\tau }^{T}\right]}^{T}$. Relying on the approximation $Q_{{\hat{c}}_{k\tau |k\tau }}\approx 0$, the observation vectors ${\left[{\underline{u}}_{h}^{T},\;{\underline{\hat{c}}}_{h|k\tau }^{T}\right]}^{T}$ (k=1,2,…) would then become time-uncorrelated, leading the user-filter to deliver close-to-minimum-variance solutions. Under the proposed formulation, however, the user needs to know the *dynamic model* that the provider uses for the corrections. For further standardization of the State Space Representation (SSR) corrections (https://www.igs.org/formats-and-standards (accessed on 18 December 2022)), Radio Technical Commission for Maritime Services (RTCM) committees may therefore request PPP-RTK providers to disclose the dynamic models underlying their SSR corrections.

A single-station PPP-RTK setup was employed to numerically demonstrate the superiority of the proposed filter over existing formulations. To emphasize the performance of our formulation, rather high correction latencies were considered, e.g., ionospheric correction packs were provided every 30 s. It was observed that the proposed filter delivers smaller estimation errors (in an RMS sense) when handling time-delayed corrections.

In the present contribution, attention was focused on the formulation of the user-filter only. Addressing open research questions such as ‘how to develop measures for assessing the approximation $Q_{{\hat{c}}_{k\tau |k\tau }}\approx 0$’, and ‘the extent to which such approximation drives the sub-optimality level of the user-filter’ are topics of future works.

## Data Availability

The GNSS data used in this contribution are freely accessible via http://saegnss2.curtin.edu.au/ldc/ (accessed on 18 December 2022) and https://cddis.nasa.gov/archive/gnss/data/ (accessed on 18 December 2022).

## Appendix A. Supplementary Proofs

**Proof of** **(7).** Combining the TU error $c_{i+1}-{\underline{\hat{c}}}_{i+1|i}=\Phi^{c}\;\left(c_{i}-{\underline{\hat{c}}}_{i|i}\right)+{\underline{w}}_{i+1}^{c}$ with its MU counterpart $c_{i+1}-{\underline{\hat{c}}}_{i+1|i+1}=\left(I-K_{i+1}^{c}A_{i+1}\right)\;\left(c_{i+1}-{\underline{\hat{c}}}_{i+1|i}\right)+K_{i+1}^{c}\;{\underline{e}}_{i+1}$ establishes a link between the two successive MU errors ${\underline{\hat{\epsilon }}}_{i}=c_{i}-{\underline{\hat{c}}}_{i|i}$ and ${\underline{\hat{\epsilon }}}_{i+1}=c_{i+1}-{\underline{\hat{c}}}_{i+1|i+1}$ as follows

(A1) $${\underline{\hat{\epsilon }}}_{i+1}=\left[\left(I-K_{i+1}^{c}A_{i+1}\right)\Phi^{c}\;\right]\;{\underline{\hat{\epsilon }}}_{i}+\underset{{\underline{l}}_{i,i+1}}{\underset{︸}{\left(I-K_{i+1}^{c}A_{i+1}\right)\;{\underline{w}}_{i+1}^{c}+K_{i+1}^{c}\;{\underline{e}}_{i+1}}}$$

where the random quantity ${\underline{l}}_{i,i+1}$ is a function of the process and measurement noises ${\underline{w}}_{i+1}^{c}$ and ${\underline{e}}_{i+1}$. The repeated application of (A1) for the remaining successive epochs i+q+1 and i+q+2 (q=1,…,j−i−q−2) gives

(A2) $${\underline{\hat{\epsilon }}}_{j}=\left[\prod_{h=i+1}^{j}\left(I-K_{h}^{c}A_{h}\right)\Phi^{c}\right]\;{\underline{\hat{\epsilon }}}_{i}+{\underline{l}}_{i,i+1,\ldots ,j},\;j>i$$

where the random quantity ${\underline{l}}_{i,i+1,\ldots ,j}$ is a function of the process and measurement noises ${\underline{w}}_{i+q}^{c}$ and ${\underline{e}}_{i+q}$ (q=1,…,i−j). Since the MU error ${\underline{\hat{\epsilon }}}_{i}$ is *uncorrelated* with the process and measurement noises of the upcoming epochs h=i+1,…,j [26], it is also uncorrelated with their linear function ${\underline{l}}_{i,i+1,\ldots ,j}$. Therefore, an application of the covariance propagation law to (A2), together with the definition $Q_{{\hat{c}}_{i|i}}:=\mathrm{Cov}\left({\underline{\hat{\epsilon }}}_{i},{\underline{\hat{\epsilon }}}_{i}\right)$, gives (7). □

**Proof of** **(8).** From the TU errors

(A3) $$c_{k\tau +q}-{\underline{\hat{c}}}_{k\tau +q|k\tau +q-1}=\Phi^{c}\left(c_{k\tau +q-1}-{\underline{\hat{c}}}_{k\tau +q-1|k\tau +q-1}\right)+{\underline{w}}_{k\tau +q}^{c},\;q=1,\ldots ,i-k\tau ,$$

follows that

(A4) $$c_{i}-{\underline{\hat{c}}}_{i|k\tau }=\Phi_{\left(i-k\tau \right)}^{c}\left(c_{k\tau }-{\underline{\hat{c}}}_{k\tau |k\tau }\right)+\sum_{h=k\tau +1}^{i}\Phi_{\left(i-h\right)}\;{\underline{w}}_{h}^{c}.$$

The application of the covariance propagation law to (A4), together with $\mathrm{Cov}(c_{k\tau }-{\underline{\hat{c}}}_{k\tau |k\tau },{\underline{w}}_{h}^{c})=0$ (h=kτ+1,…,i), gives (8). □
